# Personality-Driven Response Patterns in Accelerated iTBS: The Role of Novelty Seeking in Treatment-Resistant Depression

**DOI:** 10.1192/j.eurpsy.2026.11756

**Published:** 2026-07-31

**Authors:** C. Chiarenza, B. Araújo Cavendish, D. Mao, Y. Zhang, K. La Monica, G.-R. Wu, S. De Witte, P. Horczak, C. Geleyn, M. S. Signorelli, L. Boralli Razza, C. Baeken

**Affiliations:** 1 Psychiatry Unit, Department of Clinical and Experimental Medicine, University of Catania, Catania, Italy; 2 Ghent Experimental Psychiatry (GHEP) lab, Faculty of Medicine and Health Sciences, Department of Head and Skin, Ghent University, Ghent, Belgium; 3 3 Service of Interdisciplinary Neuromodulation, Department and Institute of Psychiatry; 4 Laboratory of Neuroscience and National Institute of Biomarkers in Psychiatry, Department and Institute of Psychiatry, University of São Paulo Medical School, São Paulo, Brazil; 5 Department of Mental Health and Addiction, ASST Fatebenefratelli Sacco, University of Milan, Milan, Italy; 6 Neuroprotection and Neuromodulation Research Group (NEUR), Center for Neurosciences (C4N), Vrije Universiteit Brussel (VUB); 7 Department of Neurology and Bru-BRAIN, Universitair Ziekenhuis Brussel (UZ Brussel); 8 Department of Psychiatry, University Hospital Brussel (UZ Brussel), Brussels, Belgium; 9 Department of Electrical Engineering, Eindhoven University of Technology, Eindhoven, Netherlands

## Abstract

**Introduction:**

Treatment-resistant depression (TRD) presents a significant clinical challenge that demands innovative and rapid therapeutic approaches. Repetitive transcranial magnetic stimulation (rTMS), particularly its accelerated variant utilizing intermittent theta burst stimulation (aiTBS), has shown promising capacity to generate swift improvements in depressive symptomatology. Despite demonstrated effectiveness, substantial interindividual variability in treatment response persists.

**Objectives:**

The present investigation examined whether personality characteristics, measured via the Temperament and Character Inventory (TCI), could predict clinical outcomes in patients undergoing aiTBS protocols.

**Methods:**

Individual participant data were aggregated from two randomized, sham-controlled aiTBS trials. Participants completed a two-week medication washout period before undergoing either active or sham aiTBS targeting the left dorsolateral prefrontal cortex across four consecutive days. Depression severity was quantified using the 17-item Hamilton Depression Rating Scale at baseline and one-week follow-up. Personality traits were assessed at baseline using the TCI questionnaire. Robust linear mixed-effects modeling was employed to evaluate associations between baseline TCI dimensions and symptomatic improvement trajectories.

**Results:**

The combined sample was of 104 individuals diagnosed with TRD (50 receiving active stimulation, 54 receiving sham). Findings revealed that elevated Novelty Seeking scores significantly moderated the rate of depressive symptom reduction across the treatment period (β = -1.70, SE = 0.73, p = 0.021). Notably, this accelerated therapeutic response manifested independently of whether participants received active or sham stimulation. Additionally, higher Novelty Seeking was associated with greater baseline depression severity (β = 2.91, SE = 1.00, p = 0.004). No additional TCI dimensions demonstrated significant predictive utility for clinical outcomes.

**Conclusions:**

These findings highlight that temperamental characteristics, specifically Novelty Seeking, may influence the rate of symptom improvement in accelerated neuromodulation protocols, irrespective of active mechanism engagement. This phenomenon could involve dopaminergic sensitivity potentiated by intensive therapeutic contexts. Incorporating personality assessment into clinical neuromodulation frameworks may help identify TRD patients predisposed to demonstrate more rapid symptomatic improvement. Validation in larger, multi-site cohorts with extended longitudinal assessment is essential to establish whether sustained remission occurs and to verify the generalizability of these findings.

**Disclosure of Interest:**

None Declared

